# Too much social media? Unveiling the effects of determinants in social media fatigue

**DOI:** 10.3389/fpsyg.2024.1277846

**Published:** 2024-07-23

**Authors:** Can Qin, Ying Li, Tian Wang, Jing Zhao, Ling Tong, Jiawei Yang, Yuyin Liu

**Affiliations:** ^1^School of Music, Jiangxi Normal University, Nanchang, China; ^2^Department of Arts Management, Xinghai Conservatory of Music, Guangzhou, China; ^3^Faculty of Humanities and Social Sciences, City University of Macau, Macau, Macao SAR, China; ^4^College of Landscape Architecture and Art, Henan Agricultural University, Zhengzhou, Henan, China; ^5^School of Music and Dance, Jiangxi University of Technology, Nanchang, China; ^6^Design College, Zhoukou Normal University, Zhoukou, Henan, China

**Keywords:** social media fatigue, fear of missing out, information overload, social anxiety, lurking

## Abstract

**Introduction:**

With the boom in social media, many people spend a lot of time on these platforms. Among them, some developed negative emotions, such as fatigue, depression, or disinterest in communicating, and used social media temporarily or permanently. Therefore, this study aims to explore the antecedents of social media fatigue, including social media helpfulness, social media self-efficacy, online subjective well-being, social comparison, compulsive social media use, privacy concerns, fear of missing out, and information overload, and to further discuss the determinants of social media fatigue on social anxiety and lurking.

**Methods:**

An online questionnaire was distributed to social media users, and 659 valid samples were obtained with the help of a purposive sampling strategy. The data was analyzed by the partial least square (PLS) method.

**Results:**

The study found that social media self-efficacy had a significant negative effect on social media fatigue; compulsive social media use, fear of missing out, and information overload had a significant positive effect on social media fatigue; and social media fatigue had a significant positive effect on social anxiety and lurking.

**Discussion:**

The research results can be used as a reference for social media marketers and internet service providers in developing business strategies.

## 1 Introduction

Today, users are becoming accustomed to using social media to send and deliver messages and video calls (Nesi et al., 2018). With the popularity of social media and the increase in user dependence, it has become a part of people’s lives (Xie et al., 2021). On the other hand, the outbreak of COVID-19 has had a huge impact on people’s lives. In response to the crisis brought about by the epidemic, many countries have adopted a series of preventive measures to avoid the spread of the virus. These measures include social distancing, remote working and learning, and postponement or cancelation of events or meetings (Ares et al., 2021). With more and more activities taking place online, social media is now an effective and important way for users to get reliable information about global pandemics and health advice (Pang, 2021).

Nowadays, tech-savvy young people make up the majority of social media users, but they often experience greater information overload in digital media environments (Pang, 2020, 2021; Liu H. et al., 2021; Xu et al., 2021). As time spent on social media increases, excessive use of social media may have physical health effects such as mental fatigue, stress, and anxiety. Research has indicated that individuals are avoiding participation in these communication services due to social media fatigue (Whelan et al., 2020; Pang, 2021). Users do not necessarily have a strong psychological quality to resist information overload, leading to subjective fatigue and withdrawal from social media use (Lee et al., 2016; Pang, 2019).

Clement (2020) pointed out that 93% of organizations have adopted social media as a tool in their marketing strategies and have generated huge advertising revenue, which is expected to grow by 28.4% by 2022. Research has further found that the use of social media in sales is positively correlated with salespeople’s customer knowledge, sales behavior, and performance (Rodriguez et al., 2016; Itani et al., 2017). Social media can help organizations collect and process various customer information, thereby enabling companies to adjust their products to suit different preferences of customers (Woodcock et al., 2011).

As social media usage continues to rise, consumers are beginning to experience social media fatigue. Since social media does not create content, social media marketing is entirely dependent on user-generated content to survive and thrive (Liu et al., 2020). Therefore, when social media fatigue leads to reduced, suspended, or discontinued usage, or lurking to use social media without delivering content, it can lead marketers to worry that brand advertising on social media is less effective. For social network services (SNS) providers, as users reduce or withdraw from social media use, they will expect lower long-term profits (Dhir et al., 2018). Scholars believe that social media fatigue has a significant negative impact on users, businesses, and service providers (Oghuma et al., 2016; Shin and Shin, 2016). Fatigue can cause users to drop out of services, resulting in lower profits for companies and service operators.

Finally, a growing body of research has highlighted the potential relationship between increased social media use and various forms of negative mental health (Luqman et al., 2017; Dhir et al., 2018; Logan et al., 2018; Abi-Jaoude et al., 2020; Pang, 2021). Previous studies have pointed out that users’ strategies in the face of social media fatigue include intentions to transfer, pause, exit, and interrupt the platform (Ravindran et al., 2014; Shin and Shin, 2016; Luqman et al., 2017). However, few researches have examined lurking as a result of social media fatigue. Therefore, this study regards lurking as a consequence of social media fatigue and explores the factors that lead to social media fatigue and the psychological and behavioral effects on users. The research purpose is to explore the determinants and consequences of social media fatigue. Thus, several research questions are proposed, including (1) the factors that cause users’ social media fatigue, (2) the impact of social media fatigue on users’ psychology, and (3) the impact of social media fatigue on social anxiety and lurking.

## 2 Literature review and hypothesis development

The primary theoretical framework for this study on social media fatigue encompasses cognitive load theory (CLT), social cognitive theory, and social comparison theory. These theories offer an in-depth understanding of the psychological and emotional factors that lead to social media fatigue. Cognitive load theory, suggests that individuals have a finite capacity for processing information (Sweller and Chandler, 1991; Sweller, 2023). In the realm of social media, users often encounter an overwhelming amount of information, resulting in cognitive overload and subsequent fatigue (Kirschner et al., 2018). This theory clarifies why information overload and compulsive use of social media are pivotal antecedents of social media fatigue (Chen et al., 2023). Additionally, social comparison theory, asserts that people assess their social and personal worth by comparing themselves to others (Festinger, 1954; Powdthavee, 2024). On social media, this frequent comparison can lead to negative self-assessments and fatigue (De Vries et al., 2023). This theory supports the inclusion of social comparison and FOMO as key antecedents in this research (Gupta et al., 2021). Lastly, this research also employed social cognitive theory which indicates an individual’s confidence in coping with life stress and achieving performance (Chou et al., 2024). This theory supports the social media self-efficacy antecedent of this research (Almulla and Al-Rahmi, 2023). The selected antecedents—social media helpfulness, social media self-efficacy, online subjective well-being, social comparison, compulsive social media use, privacy concerns, FOMO, and information overload—are grounded in these theories (Rezabeigi Davarani et al., 2023; Sweller, 2023). Collectively, they provide a solid theoretical foundation for exploring the determinants and consequences of social media fatigue (Jabeen et al., 2023). The research aims to understand how these factors contribute to fatigue and its effects on social anxiety and lurking behaviors, offering valuable insights for social media marketers and internet service providers.

### 2.1 Social media fatigue

Social media fatigue comes from the word “fatigue.” Several medical studies have suggested that fatigue is a psychosomatic response and a series of phenomena of self-evaluation and stress perception (Wijesuriya et al., 2007; Pang, 2021). Other researches define social media fatigue as a subjective and multidimensional user experience, including tiredness, annoyance, anger, disappointment, caution, loss of interest, or low need/motivation to interact with others on Social media (Ravindran et al., 2014; Zhang et al., 2016; Teng et al., 2022).

In other words, excessive and compulsive use of social media, or perceived information overload on social media, may lead to users becoming tired of social media activity, a phenomenon known as social media fatigue (Ravindran et al., 2014; Bright and Logan, 2018). Because people rely heavily on Social media to connect with others and search heavily for information about the outbreak. Users are exposed to excessive and ambiguous information on social media, resulting in fatigue (Islam et al., 2021). Additionally, scholars have argued that social media fatigue is harmful to both users and service providers (Shin and Shin, 2016). For example, if users continue to use social media, their boredom and lack of enthusiasm may lead to lower engagement (Pang et al., 2024). Furthermore, users with social media fatigue may experience discontinuous or interrupted use behavior (Luqman et al., 2017; Fu et al., 2020; Liu Z. et al., 2021). In addition, social media fatigue is closely related to the health of the mind and body. Dhir et al. (2018) and Pang (2021) indicated that it causes negative psychological effects on users, such as depression, anxiety, emotional stress, and social anxiety. In conclusion, this study proposes that determinants of social media fatigue include social media helpfulness, social media self-efficacy, online subjective well-being, social comparison, compulsive social media use, privacy concerns, fear of missing out, and information overload; the consequences are social anxiety and lurking. Next, each of these determinants and consequences is described and the research hypotheses are developed.

### 2.2 Determinants of social media fatigue

#### 2.2.1 Social media helpfulness

Today, social media (e.g., Facebook, Twitter, and Instagram) have become one of the ways users communicate with each other. They provide users with the functionality and helpfulness to engage in conversations, share ideas, form relationships, and interest groups, and develop their presence, reputation, and identity (Kietzmann et al., 2011). Social media helpfulness refers to the extent to which users receive resources and useful information from exploring social media (Bright and Logan, 2018). Users perceive social media to be useful because they satisfy needs, such as communicating with others, finding friends, keeping up-to-date, and being entertained on social media (Naranjo-Zolotov et al., 2019; Taylor et al., 2022).

Foster et al. (2010) mentioned that people use social media because of their informative value. In other words, users feel that using social media is helpful to them. In addition, Logan et al. (2018) believe that users can obtain resources and useful information from social media, and then perceive the social media’s helpfulness. Therefore, this study proposes a hypothesis.

*H1*: Social media helpfulness is negatively related to social media fatigue.

#### 2.2.2 Social media self-efficacy

Bandura (1977) defines self-efficacy as the belief that an individual can organize and perform a specific action. Also, self-efficacy is a component of social cognitive theory and can be thought of as an individual’s confidence in coping with life stress and achieving performance (Schwarzer et al., 1997; Alshahrani and Rasmussen Pennington, 2018). In addition, scholars have pointed out that people with high confidence are more likely to take action and stick with it, and they are also willing to adopt new technologies or search for useful information (Stajkovic, 2006; Hocevar et al., 2014). In short, self-efficacy affects behavior (Bandura, 1986).

Research has found that media use experience has a positive effect on self-efficacy (Eastin and LaRose, 2000). As a result, users’ perceived ability to use social media increases, and their willingness to share information increases, resulting in happier feelings (Lenhart et al., 2010). In addition, Bearden and Netemeyer (1999) proposed social media confidence as the ability of users to perceive their ability to process content on social media. Hocevar et al. (2014) argued that social media self-efficacy is the degree to which users perceive expected results to be achieved in social media. Logan et al. (2018) believe that users perceive social media self-efficacy, and their confidence will increase their willingness to use social media. In conclusion, this study suggests that social media users are less likely to experience social media fatigue when they perceive social media self-efficacy. Therefore, a hypothesis is proposed.

*H2*: Social media self-efficacy is negatively related to social media fatigue.

#### 2.2.3 Online subjective well-being

Subjective well-being is defined as a broad phenomenon that includes people’s emotional responses, domain satisfaction, and overall judgments of life satisfaction (Diener et al., 1999). It has two important components, including emotional well-being, which assesses an individual’s mood, and cognitive well-being (Russell and Daniels, 2018), which measures an individual’s life satisfaction (Verduyn et al., 2017). Emotional well-being is measured by pleasant emotions (e.g., joy, happiness, ecstasy) or unpleasant emotions (e.g., guilt, sadness, stress); cognitive well-being is measured based on one’s satisfaction with life (Di Martino et al., 2018). Changes in information technology can also affect subjective well-being. The popularity of information and communication technology in the media has improved people’s well-being (Graham and Nikolova, 2013), but it also occupies the time when people maintain relationships with friends, which indirectly has a negative impact on subjective well-being (Bruni and Stanca, 2008).

Online subjective well-being is defined as the broad range of feelings and emotions experienced by individuals using the internet and social media, such as satisfaction, well-being, and negative and positive affect (Verduyn et al., 2017; Fan et al., 2019; Kaur et al., 2021; Pang and Zhang, 2024). Huang (2016) mentioned that online subjective well-being refers to personal well-being, perceived social support, and satisfaction with online or social media life, and online social well-being has a strong impact on the continued use intention of personal social media. Previous studies have suggested that subjective well-being can be negatively affected by social media use (Gerson et al., 2016; Yao and Cao, 2017). Kaur et al. (2021) developed a research framework to examine the relationship between online subjective well-being and social media fatigue. They found that individuals who perceived higher online subjective well-being may experience lower fatigue due to their ability to properly balance and process social media communications.

Previous research examined subjective well-being as a consequence of social media use (Gerson et al., 2016). Satici and Uysal (2015) pointed out that life satisfaction and subjective well-being are negatively correlated with adverse social media use symptoms. Kaur et al. (2021) believe that users’ satisfaction and high perceived benefits from social media enable them to have higher cognitive processing ability to deal with information and content on social media, thereby experiencing low social media fatigue. In other words, social media users with high online subjective well-being experienced fewer negative phenomena, such as fatigue. In addition, previous studies have shown that social media use and personal subjective well-being are negatively correlated with negative emotions (e.g., jealousy, depression, psychological burden) (Tandoc et al., 2015; Verduyn et al., 2015), which in turn reduce life satisfaction (Frison and Eggermont, 2016) and make social media less attractive to users (De Vries and Kühne, 2015). In summary, this study proposes a hypothesis.

*H3*: Online subjective well-being is negatively related to social media fatigue.

#### 2.2.4 Social comparison

Social comparison theory (SCT) assumes that individuals may engage in two forms of social comparison, upward and downward. People assess their current abilities and ideas by comparing themselves to those who are better off (upward) or worse off (downward) (Festinger, 1954; Kim and Chock, 2015). In the absence of objective information, people have an intrinsic drive to compare themselves with others, often to gain an accurate self-evaluation. Social media provides a wealth of easily accessible information and thus can serve as a new way for people to engage in social comparisons (Burnell et al., 2019). On the other hand, if users of social media cannot have a perception of their abilities, they will compare themselves with others (Festinger, 1954; Talwar et al., 2019). Individuals compare themselves to others when confronted with information about others, such as their occupations, abilities, and achievements (Mussweiler et al., 2006). Social comparison in social media refers to the process in which individuals compare their abilities and opinions with others by browsing various information disclosed by others in the process of using social media (Yang et al., 2018). They may perceive others to be relatively better placed in the community than they are and make upward social comparisons (Latif et al., 2021).

Cramer et al. (2016) believe that comparing with others is a human tendency. Although SCT assumes that individuals can make upward and downward comparisons. However, studies exploring social media have shown that individuals tend to make more negative social comparisons, which can lead to decreased well-being, such as depressive symptoms (Faranda and Roberts, 2019). Song et al. (2019) explained that sharing content such as videos and photos on social media to positively present themselves favorably can lead others to see their positive but distorted lives. Lim and Choi (2017) found that when social comparison becomes a stressor for using social media, it may lead to emotional exhaustion in the user experience. Based on previous research findings, this study proposes a hypothesis.

*H4*: Social comparison is positively related to social media fatigue.

#### 2.2.5 Compulsive social media use

Compulsive behavior, or compulsive use, is a repetitive addiction, such as compulsive buying, overeating, or excessive use of online social media, that can have negative personal and social consequences. Compulsive use emphasizes the abnormal behavior of individuals who are unable to rationally control or regulate their daily performance (Gámez-Guadix et al., 2012; Venkatesh et al., 2019; Zhang et al., 2020). In social media research, compulsive use is often associated with internet addiction disorder (IAD) (Venkatesh et al., 2019). Unger et al. (2018) demonstrated that compulsive behavior is an addictive process in which vulnerable individuals seek escape from stress and anxiety and engage in frequent recreational and leisure activities. Despite intentional efforts to discourage or reduce compulsive behavior, it tends to persist (Gong et al., 2019).

Masur et al. (2014) found that social media addiction often leads to wasted time, reduced social connections, lower work and school performance, loss of control, and withdrawal syndrome. Compulsive use is primarily explored within a range of unhealthy physiological behaviors, including smoking or alcohol abuse, gaming addiction, and specific social media overuse (Soroya et al., 2021). Samaha and Hawi (2016) believe that smartphone addiction has a negative impact on mental health and well-being, and users with higher addiction risks experience higher perceived stress, which in turn reduces life satisfaction and academic performance. Dhir et al. (2018) used a stressor-stress-outcome (SSO) framework to explore the relationship between mental health and compulsive social media use on social media fatigue during the COVID-19 pandemic. They found that compulsive social media use significantly induced social media fatigue, which in turn led to anxiety and depression. Pang’s (2021) research also obtained similar results. Compulsive social media use is one of the major contributors to social media fatigue.

Ho et al. (2014) found that excessive internet use can lead to anxiety and depression. SNS exhaustion is a psychological consequence of excessive use of social media, resulting in low satisfaction. This phenomenon reflects individuals’ psychological responses (e.g., stress) to social media use (Maier et al., 2015). Elhai et al. (2016) found that compulsive mobile phone use affects people’s behavior and social interactions. Additionally, Dhir et al. (2018) found that compulsive social media use negatively affects cognition and performance and contributes to social media fatigue. According to previous studies, compulsive media use is positively correlated with social media fatigue (Islam et al., 2021; Mamun et al., 2021). Based on the above, this study proposes a hypothesis.

*H5*: Compulsive social media use is positively related to social media fatigue.

#### 2.2.6 Privacy concerns

With the growth of social media, online privacy is a major concern for many users. The popularity of social media and the internet has also raised concerns about privacy and security, so privacy issues are becoming more and more important. Personal privacy concerns refer to the fear that one’s personal information will be collected and misused by others, and cannot be fully protected (Stewart and Segars, 2002). Stutzman et al. (2011) believe that people who are more concerned about the improper use of personal information will engage in privacy protection behaviors. Bright and Logan (2018) mentioned that as users continue to share more personal information, privacy concerns will become their primary consideration when using social media and applications. Lee and Hsieh (2013) observed that privacy concerns are one of the components of fatigue.

Logan et al. (2018) pointed out that people with high social media self-efficacy tend to perceive the helpfulness of social media, and at the same time they will become more and more aware of privacy concerns, leading to social media fatigue. Users of social media may worry about the impact of their disclosure on their reputation in social media, leading to fatigue (Lee et al., 2019). Bright and Logan (2018) found that people who are highly concerned about privacy are prone to social media fatigue. According to past studies, high levels of privacy concerns consume social media users’ cognition and may translate into fatigue (Talwar et al., 2019; Malik et al., 2020; Kaur et al., 2021). Therefore, the hypothesis is proposed.

*H6*: Privacy concerns are positively related to social media fatigue.

#### 2.2.7 Fear of missing out

Fear is an unpleasant emotion that can damage people’s mental health. When fear is excessive, it can lead to phobias and social anxiety (Mertens et al., 2020). Fear of missing out (FoMO) is defined as worry or fear of being disconnected, absent, or missing out on experiences that others (e.g., peers, friends, family) might have or enjoy. When experiencing FoMO, people may be persistently and eagerly seeking and acknowledging the activities of others, for example, constantly checking social media content, and checking whether friends are attending parties they were not invited (Przybylski et al., 2013). The concept of FoMO applies offline, in real life, and online social media. FoMO is a constant state of mental flow. Users’ FoMO drives social media use, yet creates a sense of missing out (Przybylski et al., 2013; Tandon et al., 2021). Based on the SSO framework, FoMO is one of the important stressors that put social media users under mental and emotional stress, which in turn triggers undesirable behaviors (e.g., avoidance) (Zhang et al., 2020).

FoMO has been explored in past studies discussing social media (Whelan et al., 2020; Tandon et al., 2021). Bright and Logan (2018) found that FoMO can lead to fatigue in individuals. In addition, Tandon et al. (2021) believe that if users continue to use social media due to FoMO, they will be overloaded with information and cause fatigue. Based on the above, this study proposes a hypothesis.

*H7*: Fear of missing out is positively related to social media fatigue.

#### 2.2.8 Information overload

With the development of information technology, there are more channels for individuals to obtain a large amount of information than before. The negative results brought about by too much information have also attracted increasing attention from researchers (Luqman et al., 2017). Humans have a limited ability to process information, and information that exceeds this ability will lead to performance degradation (Hunter, 2004). Information overload is defined as a situation in which a large amount of input information exceeds the information processing capacity of an individual (Jones et al., 2004; Soto-Acosta et al., 2014; Guo et al., 2020; Islam et al., 2021). Various social media have been used as sources of crisis events and related information during COVID-19 (Islam et al., 2021). At the same time, young people frequently and excessively participate in social media activities and continuously obtain various COVID-19 information from there, which may lead to an overload of relevant information and lead to adverse psychological consequences (Liu Z. et al., 2021; Soroya et al., 2021). In addition, large amounts of information can be generated and disseminated rapidly on social media. Information overload occurs when people are exposed to more information than they can process efficiently (Maier et al., 2015; Zhang et al., 2016).

The limited capacity mode shows that individuals have limited resources to process information. Lang (2000) believed that information overload has an impact on social media fatigue. In a social media environment, users acquire vast amounts of information (Bright and Logan, 2018). However, the stress of social media-induced information overload can lead to emotional fatigue in users. When users cannot effectively integrate, absorb, and utilize too much information, it will have an impact on work, life, and interpersonal relationships (Zhang et al., 2021). In conclusion, information overload on social media may trigger user fatigue (Ravindran et al., 2014; Lee et al., 2016). Thus, the hypothesis is proposed.

*H8*: Information overload is positively related to social media fatigue.

### 2.3 Consequences of social media fatigue

#### 2.3.1 Social anxiety

Schlenker and Leary (1982) defined social anxiety as the anxiety that individuals feel when they are concerned about interpersonal evaluation when they make a specific impression on those they talk to in real or virtual social situations. Social anxiety refers to the pervasive and debilitating experience of discomfort and avoidance of interpersonal interactions due to fear of being negatively judged, rejected, or embarrassed (Panayiotou et al., 2020; Islam et al., 2021; Ran et al., 2022). Previous studies have pointed out that social anxiety is an important emotional factor, which is closely related to mobile phone addiction (Annoni et al., 2021). In addition, some studies related to the Internet have explored social anxiety (Hwang et al., 2020; Pitcho-Prelorentzos et al., 2020; Cao et al., 2022), arguing that information overload can affect emotional stress through social media fatigue and social anxiety (Pang, 2021).

In recent years, researchers have begun to explore the social anxiety of social media users. Scholars believe that when experiencing fatigue, users’ cognitive abilities decline, thereby predisposing them to inadequate regulation and control of emotions and attention, such as anxiety (Grieve et al., 2013; Fox and Moreland, 2015; Zhang et al., 2020). Social anxiety, considered a negatively reactive emotion, is a cognitive, psychological, and behavioral anxiety disorder associated with cognitive dysfunction and fatigue (Keles et al., 2020). When users experience social media fatigue, the psychological and physical effects are profound, including emotional anxiety and decreased life satisfaction and productivity (Dhir et al., 2018). Alkis et al. (2017) developed and verified the social anxiety scale of social media users, and found that undergraduate students have social anxiety caused by social media, and have higher social anxiety for SNS. Social media fatigue refers to negative emotional responses to activities on social media such as tiredness, burnout, exhaustion, frustration, and lack of interest in communicating. Based on previous literature, this study proposes the following hypothesis.

*H9*: Social media fatigue is positively related to social anxiety.

#### 2.3.2 Lurking

Social media users have shown mental and psychological deterioration due to social media fatigue (Dhir et al., 2018). Thus, users facing social media fatigue are more willing to change their status quo and existing unhealthy status (Maier et al., 2015). Lurking is associated with non-posting behavior and is defined as inactive online user behavior. They rarely post, are silent, do not participate, or have not been involved and contributed to online activities (Nonnecke and Preece, 2001). These users become social media lurkers (Sun et al., 2014). And lurking behavior can influence others to become lurkers (Zhang et al., 2021). Moreover, Rui and Stefanone (2013) believe that some users who find it difficult to adapt to the diversity of social media will overload their information, making users unable to cope effectively and choose to be lurkers. Lurking was perceived by users as a safer and easier social strategy for coping with such distress. Wisniewski et al. (2014) argue that, for social media users, lurking acts as a maladaptive countermeasure to reduce their short-term stress at the expense of increasing long-term stress.

Researchers have found that social media fatigue may be an important driver of discontinuous use intentions (Ravindran et al., 2014; Zhang et al., 2016). The variety of information and social demands on social media can overwhelm users’ processing capabilities. Users can experience fatigue after expending too much energy dealing with these demands. Lurking behaviors induced by social media fatigue include ignorance, avoidance, and withdrawal (Zhang et al., 2020). Users may use the above behaviors to escape negative emotions and fatigue (Khan, 2017). Based on the above findings, this study puts forward the following hypothesis.

*H10*: Social media fatigue is positively related to lurking.

### 2.4 Social media fatigue as a mediator

The rationale for selecting social media fatigue as a mediator in this research lies in its links to both the antecedents and outcomes of this study. Empirical studies have shown that variables such as information overload, compulsive use of social media, social comparison, and FOMO are direct contributors to social media fatigue (Przybylski et al., 2013; Bright et al., 2015; Dhir et al., 2018). Cognitive load theory posits that the cognitive burden from excessive information and compulsive behaviors leads to fatigue (Sweller, 2023), while social comparison theory suggests that social comparisons and FOMO result in emotional depletion (Powdthavee, 2024). These antecedents are specifically tied to social media fatigue, making it a more appropriate construct for the unique context of social media use. Additionally, existing research indicates that social media fatigue is a predictor of behaviors such as lurking and psychological states like social anxiety (Świątek et al., 2021; Hong et al., 2023). Consequently, social media fatigue is used as the mediator because it effectively represents the mental and emotional stress associated with social media, providing a solid theoretical and empirical foundation for examining how these antecedents lead to the identified outcomes. Hence, this research aims to examine the several indirect relationships generated from the theoretical framework with social media fatigue being a mediating variable.

**Figure 1:**
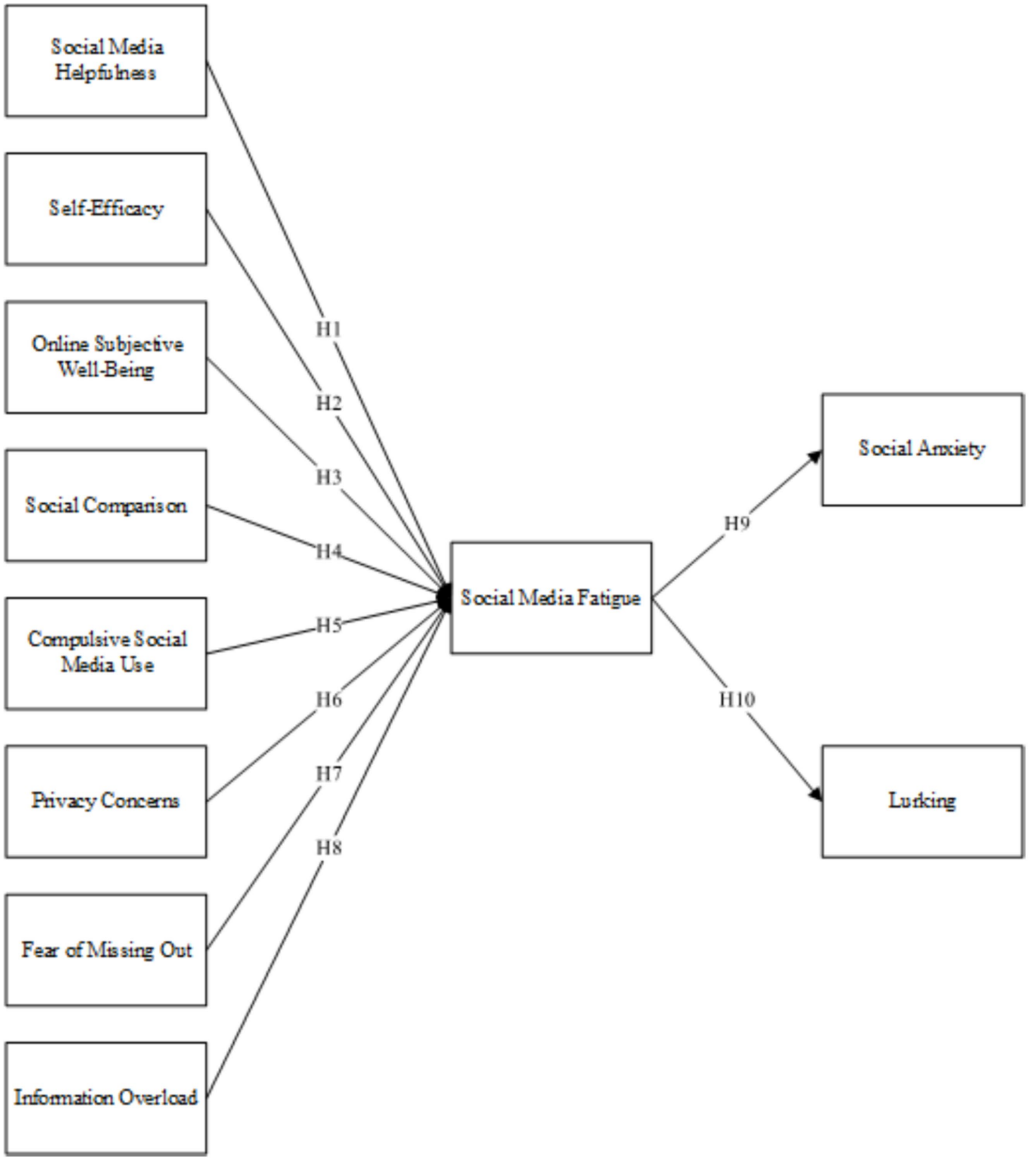
Research model.

Through a literature review, this study attempts to identify the determinants that influence social media fatigue, and its possible consequences, then formulate hypotheses and construct a research model (see Figure 1).

## 3 Research method

### 3.1 Research design

Based on the identified characteristics, the researchers defined the target population consisting of individuals who spent a significant amount of time on social media, engaged in frequent social media interactions, or exhibited behaviors indicative of compulsive social media use. The researchers employed a purposive sampling technique, which is characterized by the deliberate selection of participants possessing certain qualities that are of interest to the researcher. In this study, the researchers purposively selected participants through social media platforms known for high levels of user engagement, such as Facebook, Instagram, or Twitter based on their social media usage patterns, and targeted individuals who exhibited behaviors indicative of potential susceptibility to social media fatigue.

The study used an online questionnaire and posted the URL of the questionnaire on social media. In addition, to improve the recovery of valid questionnaires, this study also commissioned a professional academic questionnaire company to distribute. The questionnaire was distributed from February 8, 2021 to March 9, 2022. Each questionnaire was answered anonymously. Finally, a total of 659 valid questionnaires were collected. The demographics of the respondents are shown in Table 1.

**Table 1:** Demographic statistics (*N* = 659).

Measures	Items	Frequency	Percent (%)
Gender	Male	239	36.3%
	Female	420	63.7%
Age	18 or below	4	0.6%
	18–25	91	13.8%
	26–35	262	39.8%
	36–45	229	34.7%
	46–55	58	8.8%
	56–65	13	2%
	65 or above	2	0.3%
Occupation	Student	47	7.1%
	Civil servant	52	7.9%
	Service industry	254	38.5%
	Manufacturing	171	26.0%
	Financial and insurance industry	43	6.5%
	Education industry	32	4.9%
	Others	60	9.1%
Education	Elementary school	3	0.4%
	Junior high school	7	1.1%
	Senior high school	70	10.6%
	University	469	71.2%
	Master’s degree	108	16.4%
	Doctor’s degree	2	0.3%
Most used social media	Facebook	361	54.8%
	Instagram	164	24.9%
	PTT	84	12.7%
	Dcard	11	1.7%
	Twitter	12	1.8%
	Tik Tok	9	1.4%
	Weibo	6	0.9%
	Xiaohongshu	1	0.1%
	Snapchat	1	0.1%
	WeChat	3	0.5%
	LinkedIn	7	1.1%

The research questionnaire was divided into two parts, containing questions related to social media use and demographics (e.g., gender, age, occupation, education, and most used social media). Questions about social media use are based on previous research. The questions on social media helpfulness and self-efficacy were taken from Bright et al. (2015); the questions on online subjective well-being were referenced from Ahn and Shin (2013), Brunstein (1993), Chang and Hsu (2016), and Diener et al. (2015); the questions on social comparison were referenced from Gibbons and Buunk (1999), Latif et al. (2021), Reer et al. (2019), and Talwar et al. (2019); the questions on compulsive social media use are taken from Panda and Jain (2018); the questions on privacy concerns are taken from Dhir et al. (2018) and Malhotra et al. (2004); the questions on FoMO were taken from Przybylski et al. (2013); the questions on information overload were taken from Zhang et al. (2016); the questions on social media fatigue were taken from Dhir et al. (2018), Islam et al. (2021), and Whelan et al. (2020); the questions on social anxiety were taken from Alkis et al. (2017); and the questions on lurking were taken from Osatuyi (2015). The measurement scale was a seven-point Likert scale, ranging from 1 for “strongly disagree” to 7 for “strongly agree.” Respondents were asked to answer based on their own experience. Also, this study sought advice from experts to improve the quality of the questionnaire. The questionnaire is provided in the Appendix section of the research.

The research analyzed the data in two steps by employing a partial least squares (PLS) methodology. Firstly, the analysis regarding the convergent and discriminant validity of constructs was analyzed (Anderson and Gerbing, 1988; Rahardja et al., 2023). In the second step, the analysis regarding path coefficients and hypotheses was conducted. This study selected the PLS methodology because of its capability to analyze relationships (Petter et al., 2007) and complicated frameworks (Chin and Newsted, 1999; Tao et al., 2022).

## 4 Research results

### 4.1 Reliability and validity

This study applied Partial Least Squares (PLS) to test the measurement model and validate the research model. First, the reliability was tested by Composite Reliability (CR) and Cronbach’s Alpha. Hulland (1999) suggested that CR should be greater than 0.7, indicating that the measured variables are internally consistent. The CR of the latent variable in this study was between 0.780 and 0.950 (see Table 2), which was greater than the recommended value (0.7), indicating a good level and internal consistency of the measurement constructs. Hair et al. (2017) suggested that Cronbach’s Alpha is greater than 0.7, indicating that the constructs have good reliability. Table 2 shows that except for Cronbach’s Alpha for the construct “lurking,” which is less than 0.7, the others range from 0.765 to 0.934 (see Table 2), which means that the questionnaire has good reliability.

**Table 2:** Reliability and validity analysis.

Constructs	Items	Mean (SD)	Factor loadings	Cronbach’s alpha	CR	AVE
Social Media Helpfulness (HF)	HF2	5.627 (0.304)	0.516	0.765	0.788	0.569
	HF3	4.854 (1.312)	0.97			
	HF4	5.432 (1.105)	0.707			
Self-efficacy (SE)	SE1	5.822 (0.991)	0.882	0.848	0.875	0.637
	SE2	5.555 (1.079)	0.726			
	SE3	5.454 (1.152)	0.764			
	SE4	5.279 (1.187)	0.812			
Online Subjective Well-Being (OSWB)	OSWB1	4.998 (1.207)	0.889	0.901	0.928	0.812
	OSWB2	5.094 (1.164)	0.971			
	OSWB3	5.014 (1.186)	0.837			
Social Comparison (SC)	SC1	4.457 (1.463)	0.884	0.891	0.933	0.822
	SC2	4.358 (1.471)	0.943			
	SC3	4.514 (1.435)	0.891			
Compulsive Social Media Use (CSMU)	CSMU1	4.531 (1.413)	0.880	0.934	0.950	0.792
	CSMU2	4.300 (1.456)	0.911			
	CSMU3	4.015 (1.519)	0.904			
	CSMU4	4.167 (1.504)	0.893			
	CSMU5	4.581 (1.412)	0.859			
Privacy Concerns (PC)	PC1	5.024 (1.308)	0.912	0.890	0.922	0.750
	PC2	5.196 (1.349)	0.929			
	PC3	5.256 (1.286)	0.897			
	PC4	5.810 (1.197)	0.705			
Fear of Missing Out (FOMO)	FOMO1	3.924 (1.478)	0.857	0.837	0.891	0.674
	FOMO2	3.783 (1.552)	0.892			
	FOMO3	3.833 (1.535)	0.832			
	FOMO4	4.384 (1.306)	0.691			
Information Overload (IO)	IO1	4.716 (1.335)	0.820	0.808	0.875	0.641
	IO2	4.170 (1.383)	0.883			
	IO3	4.097 (1.320)	0.860			
	IO4	4.489 (1.205)	0.609			
Social Media Fatigue (SMF)	SMF1	3.947 (1.458)	0.849	0.906	0.934	0.780
	SMF2	4.059 (1.409)	0.918			
	SMF3	4.240 (1.436)	0.882			
	SMF4	4.149 (1.513)	0.883			
Social Anxiety (SA)	SA1	4.276 (1.474)	0.881	0.864	0.908	0.711
	SA2	4.436 (1.474)	0.890			
	SA3	4.144 (1.498)	0.791			
	SA4	4.029 (1.411)	0.806			
Lurking (LU)	LU1	5.124 (1.178)	0.706	0.576	0.780	0.542
	LU2	4.873 (1.168)	0.781			
	LU3	4.279 (1.376)	0.720			

SD, Standard Deviation; CR, Composite Reliability; AVE, Average Variance Extracted.

Next, this study examined convergent validity and discriminant validity. Discriminant Validity refers to the degree of correlation between different constructs. When the correlation between the constructs is low, it means that the constructs are different from each other, i.e., they have discriminant validity. The purpose of measuring convergent validity is to ensure that all questions in a construct have a high correlation with that construct. This study used PLS to test Factor Loading and Average Variance Extracted (AVE). The factor loadings ranged from 0.516 to 0.971 (see Table 2), which was greater than the recommended value (0.5) by Hair et al. (2017), indicating that the questions had convergent validity. In addition, this study had questions regarding the eight determinants of social media fatigue. One of the questions on social media helpfulness resulted in an AVE lower than Fornell and Larcker’s (1981) suggested value (0.5) and was removed. The AVE ranged from 0.542 to 0.822, indicating that the constructs had convergent validity. In addition, the correlations between the other constructs were smaller than the square root of the AVE for each construct, indicating discriminant validity (see Table 3). In addition to the Fornell-Larker Discriminant Validity, this study further tested the discriminant validity with the Heterotrait-Monotrait Ratio (HTMT). Table 4 shows that the HTMT ranged from 0.050 to 0.881, which is smaller than the value suggested by Henseler et al. (2015) (0.900), indicating that this study had discriminant validity. Table 5 further indicates the cross-loadings of the constructs. The highlighted values indicate that a cross-loading value for a specific construct will be the highest in the latent structure in comparison to other values. Hence, the cross-loadings further reaffirm a satisfactory discriminant validity for the constructs of this study.

**Table 3:** Fornell-Larker discriminant validity.

Constructs	HF	SE	OSWB	SC	CSMU	PC	FOMO	IO	SMF	SA	LU
HF	**0.754**										
SE	0.518	**0.798**									
OSWB	0.531	0.662	**0.901**								
SC	0.298	0.277	0.392	**0.907**							
CSMU	0.337	0.249	0.319	0.420	**0.890**						
PC	0.125	0.186	0.013	0.034	0.236	**0.866**					
FOMO	0.139	0.042	0.169	0.519	0.467	0.217	**0.821**				
IO	0.167	0.056	0.065	0.283	0.464	0.424	0.594	**0.800**			
SMF	0.126	−0.071	−0.049	0.185	0.408	0.347	0.508	0.741	**0.883**		
SA	0.049	−0.030	−0.063	0.258	0.348	0.356	0.545	0.583	0.636	**0.843**	
LU	0.185	0.265	0.194	0.304	0.316	0.325	0.388	0.405	0.367	0.458	**0.736**

HF, Social Media Helpfulness; SE, Self-Efficacy; OSWB, Online Subjective Well-Being; SC, Social Comparison; CSMU, Compulsive Social Media Use; PC, Privacy Concerns; FOMO, Fear of Missing Out; IO, Information Overload; SMF, Social Media Fatigue; SA, Social Anxiety; LU, Lurking; the value of the diagonal is the square root of AVE. The bold values indicate the highest square root of AVEs for each construct.

**Table 4:** Heterotrait-Monotrait ratio (HTMT).

Constructs	HF	SE	OSWB	SC	CSMU	PC	FOMO	IO	SMF	SA	LU
HF											
SE	0.881										
OSWB	0.742	0.815									
SC	0.368	0.334	0.457								
CSMU	0.382	0.321	0.359	0.461							
PC	0.267	0.238	0.050	0.068	0.257						
FOMO	0.149	0.124	0.203	0.606	0.512	0.225					
IO	0.168	0.111	0.118	0.330	0.513	0.487	0.687				
SMF	0.106	0.057	0.062	0.204	0.440	0.367	0.564	0.854			
SA	0.080	0.056	0.068	0.300	0.388	0.383	0.638	0.693	0.714		
LU	0.370	0.387	0.283	0.426	0.432	0.453	0.543	0.598	0.507	0.652	

HF, Social Media Helpfulness; SE, Self-Efficacy; OSWB, Online Subjective Well-Being; SC, Social Comparison; CSMU, Compulsive Social Media Use; PC, Privacy Concerns; FOMO, Fear of Missing Out; IO, Information Overload; SMF, Social Media Fatigue; SA, Social Anxiety; LU, Lurking.

**Table 5:** Cross-loadings.

Constructs	CSMU	FOMO	HF	IO	LU	OSWB	PC	SA	SC	SE	SMF
CSMU1	0.880	0.387	0.321	0.390	0.284	0.295	0.223	0.298	0.375	0.237	0.317
CSMU2	0.911	0.420	0.301	0.412	0.295	0.308	0.201	0.317	0.392	0.257	0.348
CSMU3	0.904	0.436	0.304	0.427	0.238	0.272	0.148	0.302	0.383	0.187	0.387
CSMU4	0.893	0.437	0.263	0.412	0.290	0.246	0.192	0.314	0.373	0.175	0.378
CSMU5	0.859	0.390	0.311	0.420	0.301	0.301	0.286	0.317	0.347	0.259	0.377
FOMO1	0.341	0.857	0.071	0.410	0.308	0.134	0.140	0.426	0.461	−0.016	0.367
FOMO2	0.364	0.892	0.099	0.440	0.299	0.149	0.144	0.474	0.516	0.032	0.382
FOMO3	0.339	0.832	0.099	0.418	0.265	0.069	0.124	0.468	0.442	−0.029	0.376
FOMO4	0.444	0.691	0.163	0.612	0.368	0.179	0.265	0.405	0.297	0.121	0.492
HF2	0.207	0.010	0.516	0.078	0.195	0.503	0.217	−0.022	0.206	0.616	−0.007
HF3	0.315	0.145	0.970	0.173	0.169	0.480	0.090	0.060	0.270	0.429	0.133
HF4	0.276	0.061	0.707	0.086	0.175	0.506	0.197	−0.007	0.273	0.621	0.048
IO1	0.450	0.489	0.172	0.820	0.407	0.076	0.458	0.479	0.234	0.106	0.593
IO2	0.477	0.538	0.166	0.883	0.301	0.097	0.323	0.503	0.257	0.038	0.686
IO3	0.346	0.522	0.153	0.860	0.327	0.054	0.331	0.515	0.244	0.014	0.650
IO4	0.152	0.317	0.004	0.609	0.264	−0.060	0.239	0.352	0.158	0.020	0.401
LU1	0.175	0.139	0.217	0.288	0.706	0.138	0.309	0.279	0.102	0.199	0.272
LU2	0.268	0.322	0.196	0.296	0.781	0.227	0.275	0.326	0.286	0.330	0.271
LU3	0.254	0.399	−0.008	0.309	0.720	0.062	0.131	0.406	0.284	0.052	0.266
OSWB1	0.296	0.206	0.468	0.088	0.167	0.889	0.011	−0.054	0.372	0.575	−0.030
OSWB2	0.296	0.132	0.500	0.041	0.184	0.971	0.009	−0.064	0.359	0.638	−0.059
OSWB3	0.308	0.163	0.549	0.091	0.210	0.837	0.039	−0.045	0.391	0.627	−0.010
PC1	0.219	0.218	0.105	0.399	0.282	−0.004	0.912	0.344	0.043	0.115	0.344
PC2	0.206	0.212	0.127	0.407	0.296	0.006	0.929	0.342	0.026	0.169	0.345
PC3	0.223	0.201	0.081	0.380	0.309	−0.004	0.897	0.336	0.036	0.160	0.303
PC4	0.162	0.064	0.147	0.241	0.241	0.096	0.705	0.147	−0.006	0.284	0.147
SA1	0.300	0.475	0.033	0.538	0.380	−0.088	0.298	0.881	0.181	−0.093	0.610
SA2	0.287	0.421	0.100	0.502	0.412	−0.026	0.368	0.890	0.179	0.035	0.550
SA3	0.335	0.511	0.038	0.473	0.372	−0.035	0.271	0.791	0.302	−0.015	0.493
SA4	0.254	0.438	−0.012	0.446	0.383	−0.061	0.260	0.806	0.226	−0.018	0.477
SC1	0.379	0.477	0.306	0.262	0.244	0.408	−0.039	0.196	0.884	0.267	0.164
SC2	0.389	0.478	0.237	0.261	0.279	0.344	0.056	0.253	0.943	0.246	0.179
SC3	0.374	0.457	0.272	0.248	0.306	0.315	0.075	0.253	0.891	0.241	0.159
SE1	0.158	−0.034	0.414	0.053	0.232	0.481	0.220	−0.019	0.197	0.882	−0.073
SE2	0.274	0.088	0.529	0.132	0.245	0.617	0.178	−0.002	0.252	0.726	0.005
SE3	0.239	0.081	0.442	0.051	0.198	0.554	0.113	−0.009	0.225	0.764	−0.029
SE4	0.271	0.112	0.470	0.045	0.225	0.669	0.093	−0.041	0.287	0.812	−0.051
SMF1	0.445	0.549	0.103	0.668	0.335	0.008	0.240	0.564	0.234	−0.108	0.849
SMF2	0.370	0.450	0.130	0.677	0.360	−0.029	0.324	0.560	0.171	−0.050	0.918
SMF3	0.297	0.370	0.100	0.620	0.318	−0.082	0.346	0.542	0.104	−0.056	0.882
SMF4	0.325	0.419	0.110	0.650	0.280	−0.073	0.320	0.579	0.138	−0.034	0.883

HF, Social Media Helpfulness; SE, Self-Efficacy; OSWB, Online Subjective Well-Being; SC, Social Comparison; CSMU, Compulsive Social Media Use; PC, Privacy Concerns; FOMO, Fear of Missing Out; IO, Information Overload; SMF, Social Media Fatigue; SA, Social Anxiety; LU, Lurking.

### 4.2 Structural equation modeling analysis

After testing the reliability and validity of the measurement model, the hypothesis testing analysis was performed on the structural model. This study used SmartPLS as the analytical tool for hypothesis testing. The main method was the explained variation (R^2^) to measure the fitness of the research model, and the standardized path coefficient and *p*-value to determine whether the hypotheses were supported.

Table 5 shows the results of the hypothesis testing. Social media self-efficacy had a negative significant effect on social media fatigue (*β* = −0.115, *p* < 0.05); compulsive social media use, FoMO, and information overload had a positive significant effect on social media fatigue (*β* = 0.108, *p* < 0.01; *β* = 0.121, *p* < 0.01; *β* = 0.612, *p* < 0.001). Therefore, H2, H5, H7, and H8 were supported. However, social media helpfulness, online subjective well-being, social comparison, and privacy concerns had no significant effect on social media fatigue (*β* = 0.090, *p* > 0.05; *β* = −0.093, *p* > 0.05; *β* = −0.057, *p* > 0.05; *β* = 0.050, *p* > 0.05). Therefore, H1, H3, H4, and H6 were not supported. Finally, social media fatigue had a positive and significant effect on social anxiety (*β* = 0.367, *p* < 0.001) and lurking (*β* = 0.636, *p* < 0.001), indicating that H9 and H10 were supported (Table 6).

**Table 6:** Direct effect analysis.

Paths		Path coefficient (β)	*T*-value	*p*-value	Result
H1	HF → SMF	0.090	1.871	0.061	Not supported
H2	SE → SMF	−0.115	2.070	0.039	Supported
H3	OSWB → SMF	−0.093	1.797	0.072	Not supported
H4	SC → SMF	−0.057	1.501	0.133	Not supported
H5	CSMU → SMF	0.108	2.864	0.004	Supported
H6	PC → SMF	0.050	1.454	0.146	Not supported
H7	FOMO → SMF	0.121	2.699	0.007	Supported
H8	IO → SMF	0.612	15.305	0.000	Supported
H9	SMF → SA	0.636	22.033	0.000	Supported
H10	SMF → LU	0.367	9.093	0.000	Supported

HF, Social Media Helpfulness; SE, Self-Efficacy; OSWB, Online Subjective Well-Being; SC, Social Comparison; CSMU, Compulsive Social Media Use; PC, Privacy Concerns; FOMO, Fear of Missing Out; IO, Information Overload; SMF, Social Media Fatigue; SA, Social Anxiety; LU, Lurking.

**Figure 2:**
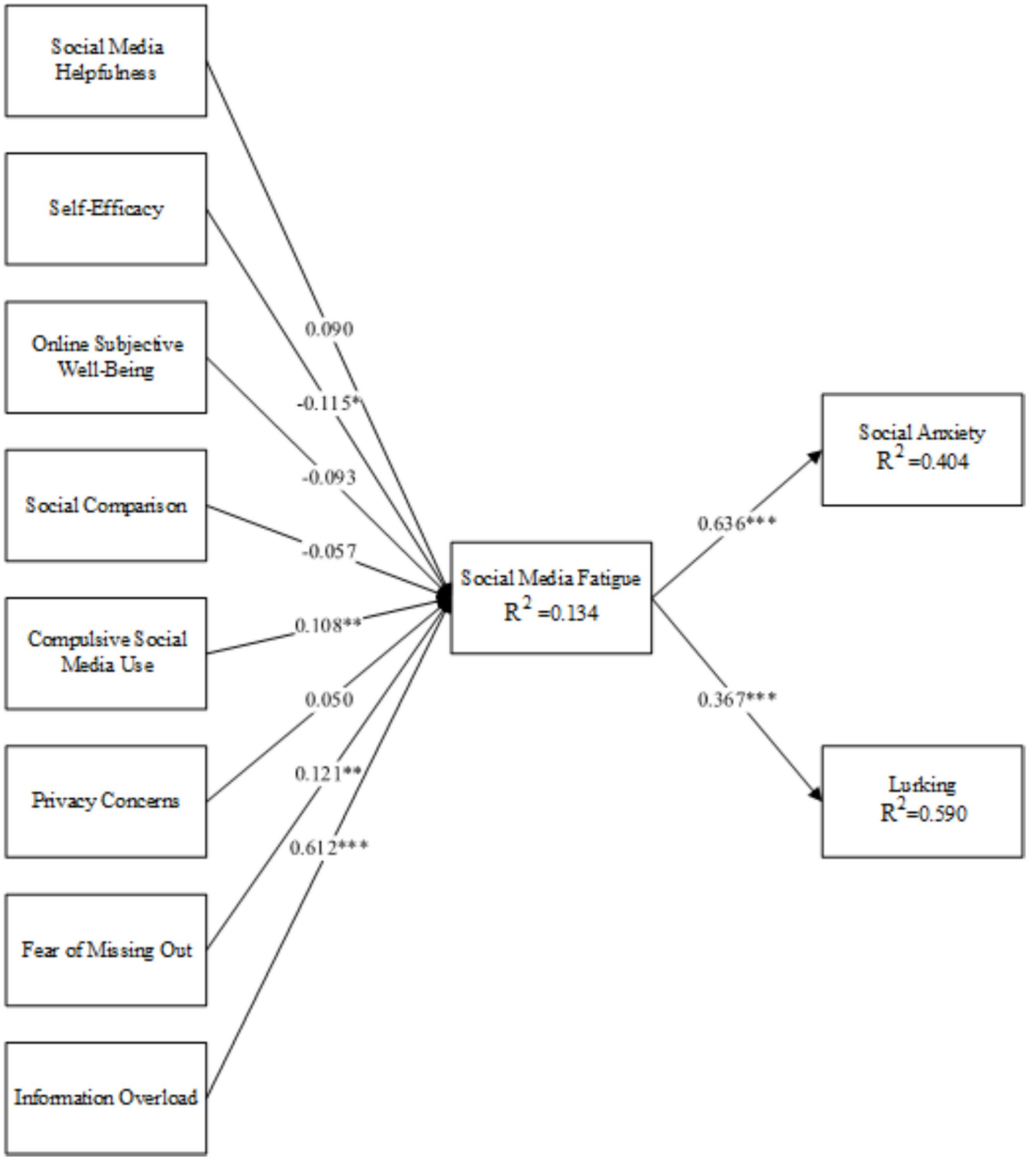
Research results.

*R*^2^ represents the ability of the dependent variable to be explained by the independent variable, or the percentage of the variance that can be explained by the exogenous variables compared to the endogenous variables. *R*^2^ is between 0 and 1. The closer it is to 1, the better the explanatory power. Figure 2 shows that the explanatory power of social media fatigue is 59.0% (*R*^2^ = 0.590), social anxiety is 40.4% (*R*^2^ = 0.404), and lurking is 13.4% (*R*^2^ = 0.134).

In addition, this study also used the results from SMART PLS to indicate indirect relationships. According to the findings indicated in Table 7. OSWB (*β* = −0.058, *T*-value = 1.764), PC (*β* = 0.031, *T*-value = 1.466), SC (*β* = −0.037, *T*-value = 1.468), and HF (*β* = 0.050, *T*-value = 1.845) did not have significant indirect relationships with SA, while having SMF as a mediator. Furthermore, SE (*β* = −0.066, *T*-value = 2.078), CSMU (*β* = 0.067, *T*-value = 2.843), FOMO (*β* = 0.078, *T*-value = 2.629), and IO (*β* = 0.390, *T*-value = 13.253) were found to significantly impact SA indirectly via SMF.

**Table 7:** Indirect relationships.

Indirect relationships	Path coefficient (β)	*T*-value	*p*-value	Result
OSWB − > SMF − > SA	−0.058	1.764	0.078	Not supported
PC − > SMF − > SA	0.031	1.466	0.143	Not supported
SC − > SMF − > SA	−0.037	1.468	0.142	Not supported
SE − > SMF − > SA	−0.066	2.078	0.038	Supported
CSMU − > SMF − > SA	0.067	2.843	0.004	Supported
FOMO − > SMF − > SA	0.078	2.629	0.009	Supported
HF − > SMF − > SA	0.050	1.845	0.065	Not supported
IO − > SMF − > SA	0.390	13.253	0.000	Supported
SC − > SMF − > LU	−0.022	1.418	0.156	Not supported
SE − > SMF − > LU	−0.038	2.064	0.039	Supported
CSMU − > SMF − > LU	0.039	2.669	0.008	Supported
FOMO − > SMF − > LU	0.045	2.436	0.015	Supported
HF − > SMF − > LU	0.029	1.822	0.068	Not supported
IO − > SMF − > LU	0.226	8.155	0.000	Supported
OSWB − > SMF − > LU	−0.034	1.721	0.085	Not supported
PC − > SMF − > LU	0.018	1.449	0.147	Not supported

HF, Social Media Helpfulness; SE, Self-Efficacy; OSWB, Online Subjective Well-Being; SC, Social Comparison; CSMU, Compulsive Social Media Use; PC, Privacy Concerns; FOMO, Fear of Missing Out; IO, Information Overload; SMF, Social Media Fatigue; SA, Social Anxiety; LU, Lurking.

Moreover, OSWB (*β* = −0.034, *T*-value = 1.721), PC (*β* = 0.018, *T*-value = 1.449), SC (*β* = −0.022, *T*-value = 1.418), and HF (*β* = 0.029, *T*-value = 1.822) did not indirectly impact LU, while having SMF as a mediator. Lastly, SE (*β* = −0.038, *T*-value = 2.064), CSMU (*β* = 0.039, *T*-value = 2.669), FOMO (*β* = 0.045, *T*-value = 2.436), and IO (*β* = 0.226, *T*-value = 8.155) were found to have significant indirect relationships with LU via SMF.

## 5 Discussion

### 5.1 Conclusion

The research purpose is to explore the determinants and consequences of social media fatigue when users use social media. Through data analysis, this research has obtained some conclusions, which are explained as follows.

First, social media self-efficacy was found to have a significant negative impact on social media fatigue. The result can be compared to an earlier study by Liu and He (2021). According to Liu and He’s (2021) study, social media has become an integral part of people’s lives. While enjoying the benefits of online communication, many young individuals are experiencing various challenges such as negative comparisons, too much information, and difficulties in interacting with others. As a result, social media fatigue (SMF) is emerging among young people. Liu and He’s (2021) study investigated the factors contributing to SMF through a questionnaire survey. Liu and He’s (2021) study identified several factors such as negative comparisons, social media self-efficacy, and information overload that significantly contributed to SMF.

Moreover, the research results showed that compulsive social media use and FoMO had significant positive impacts on social media fatigue. The results of the present study can be compared to an earlier study by Dhir et al. (2018). According to Dhir et al.’s (2018) study the rise of social media has led to increased users but also fatigue. Dhir et al.’s (2018) study investigated links between well-being and fatigue. It used a framework to examine triggers and outcomes. The data was collected from Indian adolescent users. Dhir et al.’s (2018) findings indicated that compulsive use led to fatigue, then anxiety and depression. Furthermore, the fear of missing out indirectly predicted fatigue.

On the other hand, information overload has a significant positive impact on social media fatigue. This result is similar to a previous study by Pang (2021). According to Pang’s (2021) study social media supports during pandemics like COVID-19, but its negative impacts are understudied. Pang’s (2021) study explored the effects on well-being, focusing on WeChat and information overload. Pang’s (2021) study collected the data from 566 young individuals. Pang’s (2021) study indicated that overload triggers fatigue, leading to stress and anxiety. Social media fatigue is the feeling of overwhelm, burnout, and fatigue caused by users receiving too much information from social media (Li et al., 2024). However, they also worry about not keeping up with current events and may not be able to communicate with their peers. Their chronic fear and stress of not having the same experience as others can lead to fatigue.

Consequently, the research results also showed that social media helpfulness had no impact on social media fatigue. The research results can be compared to a study conducted by Bright et al. (2015). According to Bright et al.’s (2015) study social media usage rise can cause social media fatigue. Bright et al.’s (2015) study used Lang’s model to examine information overload’s role. Bright et al.’s (2015) research explored fatigue’s antecedents including efficacy, helpfulness, confidence, and privacy concerns. According to the findings of Bright et al.’s (2015) study social media helpfulness negatively impacted social media fatigue, while privacy concerns and confidence were the top predictors of fatigue.

Furthermore, according to the present study the perceived online subjective well-being of social media users did not impact social media fatigue. This study argues that some social media users may be dissatisfied with the online community and network environment, and thus unable to use them appropriately and reduce fatigue (Zhao and Khan, 2021). The present study’s result can be compared to a study conducted by Kaur et al. (2021). According to Kaur et al.’s (2021) study scholars focus on social media’s dark impact on well-being. Kaur et al.’s (2021) study employed the limited-capacity model. Kaur et al.’s (2021) study explored the US social media users’ fatigue and collected data from Prolific Academic. Kaur et al.’s (2021) study results showed that online subjective well-being related positively to self-disclosure and social comparison, while negatively correlated with social media fatigue.

Additionally, social comparison has no significant impact on social media fatigue. This study argues that upward social comparison on social media may trigger benign jealousy and thus impact positive behavioral intentions. For example, when a friend has a superior life status on social media, it is positively related to behavioral intentions of self-enhancement and self-improvement through virtuous envy (Latif et al., 2021). Social media fatigue was not significant because comparisons with others did not cause a psychological burden. The result can be compared to a previous study by Jabeen et al. (2023). According to Jabeen et al.’s (2023) study social media’s prevalence leads to FoMO and fatigue. However, there was a lack of knowledge about their influence on users’ psychology. Jabeen et al.’s (2023) study filled this gap by examining FoMO stimuli. Jabeen et al.’s (2023) study also investigated narcissism’s impact on self-disclosure and social comparison. Jabeen et al.’s (2023) study collected data from social media users in the US. Jabeen et al.’s (2023) study results indicated that FoMO was linked to time cost and anxiety and also influenced narcissistic admiration and rivalry processes. Furthermore, Jabeen et al.’s (2023) study also indicated that social comparison positively affected fatigue.

On the other hand, the impact of privacy concerns on social media fatigue was not significant. Jang and Sung (2021) believe that although privacy concerns are related to the use of online services, highly creative users will still accept and use innovations and actively use online services. This study infers that although the website requires users to provide personal information, users who have the awareness of protecting their basic personal information will not fill in unnecessary information, and thus will not cause fatigue. Another reason is that some social media are only used by users to connect and interact with others (Malik et al., 2020). In other words, users can set their personal social media accounts to private and strictly control followers to prevent private information from being disclosed to unknown users.

Furthermore, the present research results showed that social media fatigue had a positive and significant impact on social anxiety. Social media fatigue can lead to increased social anxiety among social media users, which can be compared to previous research by Świątek et al. (2021). According to Świątek et al.’s (2021) study several interdisciplinary literatures explored social media fatigue’s correlates, including anxiety and FoMO. Świątek et al.’s (2021) study examined FoMO’s role in the anxiety-social media fatigue link. The data for Świątek et al.’s (2021) study was collected online from 264 participants, mostly women. Świątek et al.’s (2021) results indicated that higher trait anxiety is related to more intense social media fatigue. Furthermore, FoMO mediated the anxiety-social media fatigue association across dimensions.

Lastly, according to the present study, social media fatigue was found to significantly impact lurking. The result can be compared to an earlier research by Hong et al. (2023). According to Hong et al.’s (2023) study lurking surpasses interaction in social network app usage. Hong et al.’s (2023) study scrutinized lurking behavior and its drivers. Hong et al.’s (2023) study examined information refusal, browsing, and fatigue. Hong et al.’s (2023) research collected insights from 786 questionnaires and highlighted fatigue and refusal as key factors. Social media fatigue emerged as the predominant contributor to lurking.

### 5.2 Theoretical implications

The research purpose is to explore the determinants and consequences of social media fatigue. Previous studies have explored many of the determinants (e.g., self-disclosure, FoMO, social comparison, privacy concerns, information overload, and system overload) and consequences (e.g., anxiety, depression, and emotional stress) of social media fatigue (Bright et al., 2015; Lee et al., 2016; Dhir et al., 2018; Logan et al., 2018; Kaur et al., 2021; Pang, 2021; Tandon et al., 2021). However, most previous studies have focused on the factors that cause social media fatigue, but the consequences of fatigue are rarely discussed. Also, most of the previous studies discussing the consequences of social media fatigue have been about declines in social media activity, discontinuous use, and discontinuing behaviors (Luqman et al., 2017; Fu et al., 2020; Liu Z. et al., 2021).

Furthermore, by merging CLT (Sweller, 2023) with social comparison theory (Festinger, 1954; Powdthavee, 2024) and social cognitive theory (Chou et al., 2024), the research illustrates how factors like information overload (Pang, 2021) and compulsive social media use (Dhir et al., 2018) lead to cognitive exhaustion and fatigue, emphasizing the unique cognitive strain associated with digital environments. Additionally, it highlights the applicability of social comparison theory (De Vries et al., 2023) by demonstrating how frequent social comparisons and FOMO on social media platforms lead to emotional fatigue (Jabeen et al., 2023). It also signifies the importance of employing social cognitive theory (Almulla and Al-Rahmi, 2023) to indicate the relationship between self-efficacy and social media fatigue (Liu and He, 2021). Identifying social media fatigue as a mediator clarifies the indirect effects of these antecedents on outcomes such as social anxiety and lurking behaviors. Consequently, this further signifies the importance of interventions to manage these cognitive and emotional stressors. The findings promote a comprehensive framework that integrates multiple theoretical perspectives to understand the complex impact of social media on users.

This study is different from previous studies. This study uses lurking as a social media fatigue behavioral consequence, which is discussed in relatively few studies as a research direction. The research results showed a significant positive impact of social media fatigue on lurking and confirmed the relationship between these two factors. The findings contribute to research exploring social media fatigue.

### 5.3 Practical implications

The research findings have important implications for social media users, managers, and marketers. First, the implications for users. The research results show that compulsive social media use, FoMO, and information overload make users feel fatigued. Social media users should understand that compulsive use comes from their inability to restrain IAD. Also, perceptions of FoMO and information overload can directly impact an individual’s social media fatigue. Therefore, these psychological pressures can lead to fear of expressing oneself online and excessive concern about what others think of them. Second, the implications for operators and providers of social media services. This study proposes negative factors contributing to social media fatigue. Social media fatigue comes not only from human interactions but also from interactions with companies and brands (Bright et al., 2015). The purpose of users using social media is not only to establish contact with others, express personal opinions, and check news and current events but also to entertain and kill time. However, excessive use of the internet and social media leads to social media fatigue, leading to lurking. Hence, this study suggests that social media operators should strengthen the functions of social media, and provide a more concise user interface and skills or knowledge in order to improve users’ successful experience and self-confidence in the process of use, increase motivation for use, and reduce social media fatigue. Finally, the implications for marketers. The research results show that information overload and FoMO are positively related to social media fatigue. Therefore, marketers should check whether releasing too much information to users leads to information overload. Additionally, if the social media service provider can provide users with the priority to view the most interesting and favorite content, it can avoid the user’s fear of missing information, and reduce unwanted content, which can reduce information overload.

### 5.4 Research limitations and future research suggestions

Although this study took a lot of time and effort, and the process was rigorous, it was still limited by time and resources. This study is described below. First, this study explored social media fatigue without discussing specific social media. The phenomenon of social media fatigue may vary according to the characteristics of different social media or the usage habits of users. Second, this study takes social anxiety as the negative psychological impact of social media fatigue but does not explore whether social anxiety is the specific impact of social media fatigue. Therefore, future research can explore the subsequent behavior of social anxiety on social media. Third, this study adopts a cross-sectional study, which refers to data collection and investigation at a specific time point, and it cannot be confirmed that the long-term results of the study may change over time. Fourth, this study did not consider the influence of personality traits. Thus, future research can explore the characteristics of social media users and the influence of each construct on social media fatigue in more detail. Finally, most of the respondents in this study were between 26 and 45 years old. Respondents of different age groups have different habits of using social media. User experience with social media can vary based on demographics, personality traits, experience, and frequency of use. Therefore, future research can be conducted on various age groups and extend the model to various variables and different cultures or countries.

## 6 Conclusion

The study’s findings indicate that social media self-efficacy has a negative impact on social media fatigue, whereas compulsive social media use, fear of missing out (FoMO), and information overload have positive impacts. Additionally, social media fatigue is found to significantly contribute to social anxiety and lurking behaviors. These results highlight the crucial mediating role of social media fatigue, offering important insights into how various antecedents affect psychological and behavioral outcomes. This highlights the importance of targeted interventions to reduce cognitive and emotional stress among social media users. Future research should further investigate other mediating and moderating variables to deepen the understanding of these complex relationships and develop strategies that promote healthier social media usage and enhance user well-being.

## Data availability statement

The raw data supporting the conclusions of this article will be made available by the authors, without undue reservation.

## Ethics statement

Ethical review and approval was not required for the study on human participants in accordance with the local legislation and institutional requirements. Written informed consent from the patients/participants or patients/participants legal guardian/next of kin was not required to participate in this study in accordance with the national legislation and the institutional requirements.

## Author contributions

CQ: Conceptualization, Investigation, Methodology, Writing – original draft, Writing – review & editing. YiL: Methodology, Writing – original draft, Writing – review & editing. TW: Validation, Writing – original draft, Writing – review & editing. JZ: Methodology, Validation, Writing – original draft, Writing – review & editing. LT: Conceptualization, Formal analysis, Methodology, Writing – original draft, Writing – review & editing. JY: Validation, Visualization, Writing – original draft, Writing – review & editing. YuL: Conceptualization, Formal analysis, Investigation, Methodology, Writing – original draft, Writing – review & editing.
