# Supplementary material for: Too much social media? Unveiling the effects of determinants in social media fatigue
Source: Front Psychol. 2024 Jul 23;15:1277846. doi: 10.3389/fpsyg.2024.1277846 (PMC11300332; doi:10.3389/fpsyg.2024.1277846)
Supplement: Supplementary file 1 [file Table_1.docx]

Appendix

Measurement items

| **Constructs** | **Questionnaire Items** | **References** |
| --- | --- | --- |
| Social Media Helpfulness | - Social media helps me stay in touch with family and friends. - Social media helps me learn new things. - Social media helps me accomplish tasks. - Social media helps me share ideas and creations with others. | Bright et al. (2015) |
| Social Media Self Efficacy | - I find social media easy to use. - I appreciate the technological improvements brought by social media. - I believe social media is superior to other technologies (e.g., old websites/blogs). - Social media gives me more control over my life. | Bright et al. (2015) |
| Online Subjective Well-being | - In most aspects, my online social life approaches my ideal. - I am satisfied with my social life on social media platforms. - I am satisfied with my personal needs (achievement, personality, health, etc.) on social media. | Ahn & Shin (2013), Brunstein (1993), Chang & Hsu (2016), Diener et al. (2015) |
| Social Comparison | - I compare my social performance on social media with others. - I compare my achievements with others. - I compare my life situation with that of others. | Gibbons & Buunk (1999), Latif et al. (2021), Reer et al. (2019), Talwar et al. (2019) |
| Compulsive Social Media Use | - I feel uneasy when I stop using social media for a while. - I feel irritable when I cannot use social media. - Stopping the use of social media for a while makes me feel pain or despair. - I cannot control the urge to use social media. - After stopping the use of social media, I have the urge to use it again. | Panda & Jain (2018) |
| Privacy Concerns  (PC) | - I am concerned about the threat to my personal privacy on social media platforms. - I worry that the information I submit on social media platforms may be misused. - I worry that someone can find my personal information on social media. - Maintaining my privacy integrity on social media platforms is important. | Dhir et al. (2018), Malhotra et al., (2004) |
| Fear of Missing Out | - I worry that others have more valuable experiences than me. - I worry that my friends have more valuable experiences than me. - I worry when I see my friends having fun without me. - I spend too much time keeping up with current events. | Przybylski et al. (2013) |
| Information Overload | - I am often distracted by excessive information on social media. - I feel overwhelmed by the large amount of information I have to deal with on social media every day. - There is too much information about my friends on social media, so I find it bothersome to handle. - Only a small portion of the information on social media is relevant to my needs. | Zhang et al. (2016) |
| Social Media Fatigue | - The amount of information on social media makes me feel anxious and overwhelmed. - I feel mentally exhausted due to frequent use of social media. - I feel tired after using social media for a long time. - After continuous use of social media, I find it difficult to relax. | Dhir et al. (2018), Talwar et al. (2019),  Islam et al. (2021), Whelan et al. (2020) |
| Social Anxiety | - I'm afraid to express myself in case I appear awkward on social media. - I'm afraid to say something awkward on social media. - I worry that my posts will be ignored. - I find it difficult to oppose others' views on social media. | Alkis et al. (2017) |
| Lurking | - I send messages on social media without providing my personal information. - I add some friends just to keep up with their lives. - I register on some social media platforms just for social or gossip news. - I maintain registration on some social media platforms to create online accounts with other organizations (e.g., using Google/Facebook accounts to log in to other websites). | Osatuyi (2015) |
